# Maternal antioxidant treatment prevents the adverse effects of prenatal stress on the offspring's brain and behavior

**DOI:** 10.1016/j.ynstr.2020.100281

**Published:** 2020-11-29

**Authors:** H. Scott, T.J. Phillips, Y. Sze, A. Alfieri, M.F. Rogers, V. Volpato, C.P. Case, P.J. Brunton

**Affiliations:** a School of Clinical Sciences, University of Bristol, Learning & Research Building, Southmead Hospital, Bristol, BS10 5NB, UK; b Division of Neurobiology, The Roslin Institute, University of Edinburgh, Easter Bush, Midlothian, EH25 9RG, UK; c Centre for Discovery Brain Sciences, University of Edinburgh, Hugh Robson Building, George Square, Edinburgh, EH8 9XD, UK; d Intelligent Systems Laboratory, University of Bristol, Merchant Venturers Building, Woodland Road, Bristol, BS8 1UB, UK; e UK Dementia Research Institute, Cardiff University, Hadyn Ellis Building, Maindy Road, Cardiff, CF24 4HQ, UK; f Zhejiang University-University of Edinburgh Joint Institute, Zhejiang University School of Medicine, International Campus, Haining, Zhejiang, 314400, PR China

**Keywords:** Anxiety, Basolateral amygdala, Corticosterone, GABA receptors, Hippocampus, microRNAs, Oxidative stress, Placenta, Prenatal stress, Sex differences

## Abstract

Maternal exposure to stress during pregnancy is associated with an increased risk of psychiatric disorders in the offspring in later life. The mechanisms through which the effects of maternal stress are transmitted to the fetus are unclear, however the placenta, as the interface between mother and fetus, is likely to play a key role. Using a rat model, we investigated a role for placental oxidative stress in conveying the effects of maternal social stress to the fetus and the potential for treatment using a nanoparticle-bound antioxidant to prevent adverse outcomes in the offspring.

Maternal psychosocial stress increased circulating corticosterone in the mother, but not in the fetuses. Maternal stress also induced oxidative stress in the placenta, but not in the fetal brain. Blocking oxidative stress using an antioxidant prevented the prenatal stress-induced anxiety phenotype in the male offspring, and prevented sex-specific neurobiological changes, specifically a reduction in dendrite lengths in the hippocampus, as well as reductions in the number of parvalbumin-positive neurons and GABA receptor subunits in the hippocampus and basolateral amygdala of the male offspring. Importantly, many of these effects were mimicked in neuronal cultures by application of placental-conditioned medium or fetal plasma from stressed pregnancies, indicating molecules released from the placenta may mediate the effects of prenatal stress on the fetal brain. Indeed, both placenta-conditioned medium and fetal plasma contained differentially abundant microRNAs following maternal stress, and their predicted targets were enriched for genes relevant to nervous system development and psychiatric disorders.

The results highlight placental oxidative stress as a key mediator in transmitting the maternal social stress effects on the offspring's brain and behavior, and offer a potential intervention to prevent stress-induced fetal programming of affective disorders.

## Highlights

• Social stress in pregnancy induces oxidative stress but is prevented by antioxidant.
• Prenatal stress induces behavioural, neuroanatomical and neurochemical changes.
• Maternal antioxidant treatment prevents stress-induced effects in the offspring.
• Maternal stress alters the balance of microRNAs secreted from the placenta.
• Placental oxidative stress mediates maternal social stress effects on the offspring.

## 1 Introduction

Stress experienced during pregnancy is associated with an increased risk of psychiatric disorders, such as anxiety and depression, in the offspring in later life (King et al., 2012; Schuurmans and Kurrasch, 2013; Graignic-Philippe et al., 2014). In rodent models, maternal psychological stress during pregnancy gives rise to behavioral changes in the offspring, including heightened anxiety-like behavior, cognitive deficits and abnormal social behaviors (Fride and Weinstock, 1988; Brunton and Russell, 2010; Brunton, 2013; Maccari et al., 2014; Grundwald et al., 2016).

Male and female offspring are often differently affected by maternal psychological stress. For example, male, but not female rats, display increased anxiety-like behavior following maternal exposure to repeated social stress (Brunton and Russell, 2010) or restraint (Zuena et al., 2008; Iturra-Mena et al., 2018). Furthermore, elevated maternal cortisol during pregnancy is associated with increased internalizing behavior at 24 months of age in girls, but not in boys (Graham et al., 2019).

The basolateral amygdala and hippocampus play key roles in regulating anxiety-like responses to stress (McEwen et al., 2016; Zhang et al., 2018). Structural changes within these brain regions are observed in response to prenatal stress in rodents, including alterations in the number of neurons/glia, dendritic length and dendritic complexity (Vyas et al., 2002; Mitra et al., 2005; Kraszpulski et al., 2006; Mychasiuk et al., 2012; Bock et al., 2014; Soares-Cunha et al., 2018); and changes in amygdala volume have been reported in the children of women that experienced prenatal distress (Acosta et al., 2019). Furthermore, prenatal stress can result in disrupted inhibitory signalling in the brain, reflected by a reduction in the proportion of hippocampal parvalbumin-positive GABAergic interneurons in rodent offspring (Lussier and Stevens, 2016) and changes in the expression of GABA_A_ receptor subunits in the amygdala and hippocampus (Laloux et al., 2012; Nejatbakhsh et al., 2018). These findings indicate that prenatal stress can lead to disrupted GABA signalling in brain regions that regulate anxiety-like behaviors, which is important given GABAergic dysfunction is implicated in the pathophysiology of psychiatric disorders, for which prenatal stress increases risk (Nikolaus et al., 2010; Fine et al., 2014; Tovote et al., 2015; Selten et al., 2018). Although we have demonstrated maternal social stress results in increased anxiety-like behavior in the male offspring (Brunton and Russell, 2010), it is not known whether this is associated with GABAergic dysfunction.

Despite much research into the consequences of maternal stress in the offspring, it remains unclear how the sex-specific effects of maternal psychological stress are transmitted from the mother to the fetus(es). The placenta, as the interface between mother and fetus, is likely to play a key role in maternal programming of fetal development (O'Donnell et al., 2009; Brunton and Russell, 2011; Bronson and Bale, 2016). Indeed, maternal exposure to different stressors affects placental growth and morphology (Hewitt et al., 2006; Walker et al., 2013), metabolism and endocrine function (Mairesse et al., 2007; Fowden et al., 2009; Bonnin et al., 2011; O'Donnell et al., 2012), gene expression (Mairesse et al., 2007; Mueller and Bale, 2008; Gheorghe et al., 2010; Ponder et al., 2011; Jensen Pena et al., 2012; O'Donnell et al., 2012; Howerton et al., 2013) and the epigenetic profile (Jensen Pena et al., 2012; Palma-Gudiel et al., 2018) – characteristics that have been associated with altered fetal neurodevelopment and disease (Hamdan et al., 2009; Howerton et al., 2013; Howerton and Bale, 2014; Green et al., 2015; Perez-Garcia et al., 2018). In addition, growing evidence indicates that excessive oxidative stress is associated with adverse pregnancy outcomes, which in turn may contribute to fetal programming of disease in later life (Rakers et al., 2017). Indeed, we previously showed that using an antioxidant to block the increase in placental oxidative stress induced by maternal hypoxia, prevents abnormal neurobiological changes in the offspring (Phillips et al., 2017). However, it is not yet known whether maternal social stress induces oxidative stress in the mother, placenta and/or fetuses, or what role, if any; this may play in mediating the impact of maternal stress on the offspring's brain and behavior.

Therefore, here, using a rodent model of maternal psychosocial stress during pregnancy, we investigated the role of the placenta and oxidative stress in mediating the effects of prenatal stress on the fetus. After first establishing a role for maternal social stress in inducing oxidative stress in the placenta, we tested whether: (i) the sex-specific behavioral effects of maternal social stress are associated with sex-specific neurobiological changes in the offspring's brain, particularly focussing on altered inhibitory GABAergic signalling in the amygdala and hippocampus; (ii) administration of an antioxidant to reduce oxidative stress in the placenta could prevent these behavioral and neurobiological effects of maternal stress in the offspring; and (iii) microRNAs are differentially secreted from the placenta in response to maternal social stress. Our overarching aim was to better understand the factors involved in mediating the changes in the offspring's brain and behavior following maternal social stress exposure in pregnancy.

## 2 Materials & methods

### 2.1 Animals

All animal procedures were conducted in accordance with the UK Animals (Scientific Procedures) Act 1986 and the European Directive (2010/63/EU), with institutional approval from the University of Edinburgh Animal Welfare and Ethical Review Body.

Female Sprague-Dawley rats (initially weighing 240–250g; Charles River, Kent, UK) were maintained on *ad libitum* standard rat chow (Teklan, 2014 diet, Harlan Laboratories, UK; supplemented 1:1 with Teklan, 2019 diet throughout pregnancy and lactation) and filtered tap water under a 12:12-h light-dark cycle (lights on at 07:00h). Following ≥7d acclimatisation to the facility, virgin female rats were housed overnight with a sexually experienced male. Mating was confirmed by the presence of a semen plug in the breeding cage the following morning (designated day 1 of pregnancy). Rats were group housed (4–6/cage) in individually-ventilated cages until gestational day (GD) 16 and singly thereafter.

### 2.2 Maternal social stress paradigm and drug treatment

On GD16, pregnant rats were weighed and 4% lidocaine cream was applied topically to the tail before administration of either 0.9% sterile saline (vehicle; 0.34 ml/kg) or 90 μg/kg nanoparticle-bound mitoquinone (MitoQ-NP; 0.34 ml/kg) i.v. into the tail vein. The nanoparticle-bound MitoQ enters the placenta but does not cross it (Phillips et al., 2017). 1h later, half of the vehicle and MitoQ-NP-treated rats were exposed to 10 min social stress, with the remainder serving as non-stressed controls. Social stress was used as this is an ethologically relevant stressor for rats and it may better reflect the types of stress that pregnant women may experience, which generally involve a social component (Bjorkqvist, 2001; Brunton, 2013). Social stress was induced using a modified resident-intruder paradigm as previously described (Brunton and Russell, 2010). Briefly, pregnant rats (‘intruder’) were transferred to an adjacent room and exposed to a different unfamiliar aggressive lactating rat (‘resident’ between days 1–7 of lactation) daily for 10 min/day on GD16-20 in the resident's open-top home cage. A different ‘resident’ rat was used on each day.

### 2.3 Animal experiments

#### 2.3.1 Experiment 1: Effects of prenatal stress on the mother, fetus and placenta at GD20

Pregnant rats (n = 28) underwent vehicle/drug treatment with or without social stress exposure (as above, see section 2.2). Immediately after the final social stress exposure on GD20, pregnant rats and fetuses were killed by conscious decapitation. Maternal trunk blood was collected into chilled tubes containing 5% (w/v) EDTA. Fetal trunk blood was collected into EDTA-coated microvette tubes (Sarstedt, Germany), pooled by sex and litter. Plasma was separated by centrifugation at 4 °C, frozen on dry ice and stored at −20 °C until further analysis. Placentae (n = 3/litter) were collected and cultured (see section 2.9) to generate conditioned media. Additional placentae, together with maternal and fetal samples of brain and liver were also collected and frozen on dry ice before storage at −80 °C for oxidative stress analysis (see section 2.6).

#### 2.3.2 Experiment 2: Effects of prenatal stress on offspring brain and behavior

A separate cohort of pregnant rats (n = 32) underwent the same drug treatment and social stress exposure (as above, see section 2.2) and were allowed to give birth (typically on GD22.5–23). Pups were weaned on postnatal day (P) 23 and housed in same sex groups by litter (4–6 females, 3–5 males). At P30, a cohort of offspring (n = 6–7 males and n = 6–7 females per treatment group, from separate litters) were deeply anaesthetised with 3% isoflurane in 1000 ml/min O_2_, before cardiac perfusion-fixation. Following fixation, brains were removed, cryoprotected, frozen on dry ice and stored at −80 °C until immunohistochemistry processing (see section 2.8). Littermates were used for the behavioral experiments (see section 2.7).

### 2.4 Preparation of MitoQ-loaded nanoparticles

Antioxidant MitoQ (Kelso et al., 2001) was loaded onto γ-PGA-Phe nanoparticles (Kim et al., 2009) to produce MitoQ-NP as previously described (Phillips et al., 2017). MitoQ-NP was used at a final dose of 0.5 μM MitoQ. Rats received a single injection of either saline or MitoQ-NP on day 16 of pregnancy, 1h prior to the first social stress exposure (see section 2.2).

### 2.5 Determination of plasma corticosterone concentrations

Plasma corticosterone concentrations in maternal plasma and pooled fetal plasma (collected in Experiment 1) were determined in duplicate using a commercially available radioimmunoassay kit (MP Biomedicals, Germany; sensitivity, 7.7 ng/ml; intra-assay variation, 4.4–10.3%).

### 2.6 Quantification of reactive oxygen species

Reactive oxygen species (ROS) levels were measured in fetal, maternal and placental tissues (from Experiment 1) using the 2′,7′-dichlorofluorescein diacetate (DCFDA) (Sigma-Aldrich) assay. Cryostat-cut sagittal sections (10 μm) were exposed to 20 μM DCFDA solution in HBSS at 37 °C in a humidifying chamber. After counter-staining with DAPI, sections were immediately imaged using a confocal microscope (excitation: 495 nm, emission: 529 nm). Fluorescence levels of DCF were quantified using Image-Pro Premier 9.2 (Media Cybernetics, USA).

### 2.7 Behavioral tests in the offspring

The offspring (one male and one female/litter; n = 8/group/sex; from Experiment 2) were tested for anxiety-like behavior at 9 weeks of age using the light-dark box (Crawley, 1981), followed by the elevated plus maze 4 days later. These tests have been used extensively in rodents as assays of anxiety-like behavior (Lezak et al., 2017) and have been validated with anxiolytic drugs (Pellow et al., 1985; Costall et al., 1989). Importantly, we previously demonstrated an anxiety-like phenotype in the male prenatally stressed offspring of mothers exposed to social stress during pregnancy, using the elevated plus maze (Brunton and Russell, 2010). Both tests were perfomed in the same room under the same lighting conditions (ambient light = 330 lux).

#### 2.7.1 Light-dark box

The light-dark box consisted of a transparent box and lidded opaque black box connected by an opening, as described previously (Grundwald and Brunton, 2015). Rats were placed into the dark chamber and allowed to explore freely for 5 min. The trials were recorded with an infrared camera and the time spent in each compartment, the latency to enter the light box and the distance travelled was scored using EthoVision XT software v12 (Noldus, Wagenigen, The Netherlands).

#### 2.7.2 Elevated plus maze

The elevated plus maze consisted of 2 open arms (600 × 100 mm with 5 mm lip), located opposite each other and intersected at a central point (100 × 100 mm) with 2 closed arms (600 × 100 mm with 350 mm walls), elevated ca. 75 cm above the floor (Grundwald and Brunton, 2015). Rats were placed at the intersection, facing an open arm, and were allowed to explore freely for 10 min. The number of open arm entries and total arm entries was quantified using EthoVision XT software v12. For both tasks, the mazes were thoroughly cleaned with 70% ethanol and allowed to air-dry between each trial and males and females were tested on separate days.

### 2.8 Immunohistochemistry on offspring brains

Brains (n = 6/treatment group from different litters generated in Experiment 2) were processed for immunohistochemistry. Coronal cryostat sections (12 μm) including the hippocampus (CA1, CA2, CA3) and the basolateral amygdala were mounted as contiguous triplicates. Sections were fixed in cold methanol (−20 °C), blocked with 5% goat serum, 0.3% Triton X-100 in phosphate buffered saline (PBS) for 2h at 4 °C, followed by an overnight incubation at 4 °C in primary antibody against either parvalbumin (1:500, ab11427; abcam), MAP2 (1:500, ab32454; abcam), GABA Aα1 (1:500, ab33299; abcam), GABA Aα2 (1:500, ab193311; abcam) or GABA B1 (1:500, ab55051; abcam) in PBS with 1% bovine serum albumin (BSA), 0.3% Triton X-100. Sections were incubated with Alexa Fluor 555 anti-rabbit IgG, Alexa Fluor 488 anti-mouse IgG or Alexa Fluor 568 anti-mouse IgG secondary antibodies (Thermo Fisher Scientific) at 1:500 for 2h at 4 °C. Vectashield Mounting Medium with DAPI (Vector Laboratories, USA) was used to mount coverslips.

#### 2.8.1 Analysis of immunohistochemistry on offspring brains

The intensity of the immunostaining was analysed as previously described (Phillips et al., 2017). Briefly, 6 brains/group were immunostained as previously described and imaged using a LASX (Leica) widefield microscope or a SP5II (Leica) confocal microscope at 40x magnification using oil. 9 sections per brain were imaged and 5 images were taken per brain region in each hemisphere. Images were converted to greyscale and the total pixel number was determined using a corrected macro for ImageJ. Background staining was found using relative isotype controls. The background was subsequently subtracted from the total pixel number. Dendrite lengths were measured in sections stained with MAP2 using ImageJ.

### 2.9 Preparation of placenta-conditioned media

Whole fresh rat placentae (isolated from GD20 rats in Experiment 1) were incubated individually at 21% O_2_, 5% CO_2_ and 37 °C in neuronal culture medium (Gibco Neurobasal medium with 1x Gibco B-27 supplement, 2 mM L-glutamine and 250 μM penicillin-streptomycin; all Thermo Fisher Scientific). After 24h, the media around the explants was collected, syringe filtered (0.2 μm), frozen on dry ice and stored at −80 °C until further use.

### 2.10 Cortical cultures

Cortical cultures were prepared from dissociated rat embryonic day 18 cortical tissue and grown on glass coverslips as described previously (Curtis et al., 2014) in neuronal culture medium (see section 2.9). Placental-conditioned medium or fetal plasma (n = 5/treatment group, each originating from a different litter collected in Experiment 1) was applied to the cortical cultures for 6 days starting from day 12 *in vitro*. Exposures were performed in triplicate with 3 different sets of cortical cultures.

### 2.11 Immunocytochemistry on cortical cultures

Cortical cultures were fixed in supercold methanol (−20 °C) and blocked with 5% BSA, 5% normal goat serum in PBS for 30 min. Cultures were then incubated at 4 °C overnight with primary antibodies against MAP2 or the GABA receptors (as before, see section 2.8). Sections were probed with secondary antibodies, Alexa Fluor 488 anti-rabbit IgG or Alexa Fluor 488 anti-mouse IgG (Thermo Fisher Scientific, diluted 1:500) for 2h at room temperature under minimal light conditions, washed with PBS and mounted in DAPI mounting media. Five images/coverslip were captured on a confocal microscope (SP2-AOBS, Leica). Dendrite lengths were measured using ImageJ. For the analysis of receptors, fluorescent images were taken at 64x magnification (with oil) using a Leica SP5II fluorescence microscope after excitation at 488 nm. Using ImageJ, images were converted to RGB files and the mean grey value (representing the relative intensity of the staining) of each image was measured, as described previously (Phillips et al., 2017).

### 2.12 Analysis of microRNAs in placental-conditioned medium and fetal plasma

The microRNA profile of the placental-conditioned medium and fetal plasma (from Experiment 1) was analysed as described previously (Phillips et al., 2017). Briefly, total RNA was extracted from 200 μl placenta-conditioned medium or 100 μl fetal plasma using the miRNeasy Mini Kit and the miRNeasy Serum/Plasma Kit (Qiagen, Germany). microRNA expression levels were analysed using the nCounter Rat v1.5 miRNA Expression Assay (NanoString Technologies, USA), which detects over 400 different species-specific microRNAs. Undiluted samples (3 μl) were hybridised with barcoded probes and immobilised on an nCounter Cartridge. Barcode signals were counted using the nCounter Digital Analyser.

### 2.13 Bioinformatic analyses

NanoString nCounter data was analysed using a pipeline described previously (Phillips et al., 2017). RUVSeq (Risso et al., 2014) was used to adjust the counts to account for unwanted variation and then edgeR (Robinson et al., 2010) to predict differentially abundant microRNAs from the adjusted counts. miRNAs were classed as significant differentially secreted miRNAs if *p* < 0.05. Predicted targets of differentially abundant microRNAs were derived from TargetScanHuman v7.0 (Agarwal et al., 2015) (Total Context Score < −0.2) and subjected to gene ontology analysis using DAVID 6.8 (Huang da et al., 2009a; b). Enrichment analyses were performed in R/Bioconductor using the Fisher's exact test and investigating enrichment only. Venn diagrams were produced using Venny 2.1 (http://bioinfogp.cnb.csic.es/tools/venny/index.html).

Gene set association analysis across neuropsychiatric traits was performed with two polygenic approaches, partitioned LD score regression (LDSC) method (Finucane et al., 2018) and MAGMA method (de Leeuw et al., 2015). LDSC tests gene set enrichment in heritability from genome wide association studies (GWAS) summary statistics. MAGMA performs gene set enrichment analysis based on GWAS summary statistics while also accounting for LD structure between SNPs, gene size, gene density and mean sample size. For each GWAS summary statistics we excluded SNPs with MAF <1%, set a window of±25 kb around each gene to compute gene-level association statistic by averaging GWAS P-values of the identified SNPs, and used the European reference panel from the phase 3 of the 1000 genomes project (Auton et al., 2015) as the reference population. The multiple test correction significance threshold was set to 0.05 divided by the number of tested gene sets.

### 2.14 Data availability

Raw and processed data files generated from NanoString analysis have been deposited in NCBI's Gene Expression Omnibus (Edgar et al., 2002) and are accessible through GEO accession number GSE130573.

### 2.15 Data and statistical analyses

In each case, analysis of behavior and microscopy images was performed with the experimenter blind to treatment group. Data are presented as group means ± s.e.m. Two-way ANOVAs were used to test for main effects of prenatal status (maternal stress exposure or control pregnancy) and drug treatment (vehicle or MitoQ-NP) and for prenatal status × drug interaction effects and the results of these are presented in Table 1. Where the ANOVA indicated a significant main effect, post-hoc analysis using Tukey's or Student-Newman-Keuls test for multiple comparisons (see the figure legends for details) was used to identify differences between groups. In each case, p ≤ 0.05 was considered statistically significant. Group differences are indicated by symbols on the individual graphs (see figure legends for details). Statistical analyses were performed in Prism 6.0 (GraphPad, USA) and SPSS 21.0 (IBM Corp., USA).

**Table 1:** Results of two-way ANOVA analyses for data presented in Fig. 1, Fig. 2, Fig. 3, Fig. 4, Fig. 5, Fig. 6. Main effects of prenatal stress exposure, MitoQ-NP and interactions between the two factors were tested and the *F* and *p* values are presented. Where significant main effects were detected, post-hoc multiple comparison tests were performed and any significant differences between treatment groups are indicated by symbols in the relevant figures.

	Prenatal stress effect		MitoQ-NP effect		Interaction	
**Figure**	*F value*	*p value*	*F value*	*p value*	*F value*	*p value*
1a	F(1,24) = 25.2	**<0.001**	F(1,24) = 1.16	0.29	F(1,24) = 0.05	0.83
1b	F(1,23) = 0.91	0.31	F(1,23) = 0.04	0.84	F(1,23) = 0.29	0.65
1c	F(1,24) = 2.34	0.14	F(1,24) = 0.03	0.86	F(1,24) = 2.98	0.097
2a	F(1,8) = 30.64	**0.001**	F(1,8) = 5.27	0.051	F(1,8) = 12.97	**0.0019**
2b	F(1,8) = 15.10	**0.005**	F(1,8) = 15.33	**0.004**	F(1,8) = 16.07	**0.0010**
2c	F(1,8) = 14.57	**0.005**	F(1,8) = 7.16	**0.028**	F(1,8) = 9.74	**0.0048**
2d	F(1,8) = 7.52	**0.025**	F(1,8) = 2.22	0.175	F(1,8) = 5.56	**0.0024**
2e	F(1,8) = 77.72	**<0.0001**	F(1,8) = 0.35	0.569	F(1,8) = 31.69	**<0.0001**
2f	F(1,8) = 0.29	0.605	F(1,8) = 0.42	0.533	F(1,8) = 0.24	0.8676
3a	F(1,27) = 4.50	**0.043**	F(1,27) = 1.52	0.23	F(1,27) = 2.78	0.107
3b	F(1,26) = 7.57	**0.011**	F(1,26) = 0.83	0.37	F(1,26) = 3.36	0.078
3c	F(1,27) = 0.03	0.86	F(1,27) = 0.66	0.42	F(1,27) = 0.24	0.63
3d	F(1,28) = 0.03	0.86	F(1,28) = 0.43	0.52	F(1,28) = 0.27	0.61
4a - CA1	F(1,20) = 6.06	0.230	F(1,20) = 2.52	0.273	F(1,20) = 0.03	0.86
4a - CA2	F(1,20) = 1.14	0.299	F(1,20) = 2.81	0.11	F(1,20) = 0.21	0.653
4a - CA3	F(1,20) = 7.38	**0.013**	F(1,20) = 0.68	0.42	F(1,20) = 21.3	**0.0002**
4a - BLA	F(1,20) = 38.0	**<0.0001**	F(1,20) = 0.004	0.94	F(1,20) = 0.75	0.101
4b - CA1	F(1,20) = 7.59	**0.012**	F(1,20) = 3.94	0.061	F(1,20) = 7.48	**0.0015**
4b - CA2	F(1,20) = 21.5	**0.0002**	F(1,20) = 23.2	**0.0001**	F(1,20) = 15.2	**<0.0001**
4b - CA3	F(1,20) = 3.02	0.0975	F(1,20) = 41.01	**<0.0001**	F(1,20) = 18.39	**<0.0001**
4b - BLA	F(1,20) = 45.39	**<0.0001**	F(1,20) = 8.10	**0.01**	F(1,20) = 19.50	**<0.0001**
4c - CA1	F(1,20) = 0.91	0.351	F(1,20) = 1.22	0.282	F(1,20) = 0.65	0.431
4c - CA2	F(1,20) = 0.07	0.788	F(1,20) = 0.49	0.491	F(1,20) = 0.03	0.875
4c - CA3	F(1,20) = 0.66	0.427	F(1,20) = 0.84	0.371	F(1,20) = 0.50	0.488
4c - BLA	F(1,20) = 22.07	**0.019**	F(1,20) = 2.82	0.109	F(1,20) = 3.91	0.062
4d - CA1	F(1,20) = 21.58	**0.0002**	F(1,20) = 1.04	0.320	F(1,20) = 9.12	**0.0005**
4d - CA2	F(1,20) = 28.95	**<0.0001**	F(1,20) = 2.77	0.112	F(1,20) = 13.61	**<0.0001**
4d - CA3	F(1,20) = 28.55	**<0.0001**	F(1,20) = 0.06	0.804	F(1,20) = 10.79	**0.0002**
4d - BLA	F(1,20) = 11.37	**0.003**	F(1,20) = 7.35	**0.0134**	F(1,20) = 6.90	**0.0023**
5a - CA1	F(1,20) = 64.45	**<0.0001**	F(1,20) = 0.006	0.941	F(1,20) = 12.55	**0.0022**
5a - CA2	F(1,20) = 153.3	**<0.0001**	F(1,20) = 0.034	0.855	F(1,20) = 25.76	**0.0002**
5a - CA3	F(1,20) = 7.14	**0.0146**	F(1,20) = 0.38	0.544	F(1,20) = 1.27	0.349
5a - BLA	F(1,20) = 158.2	**<0.0001**	F(1,20) = 1.16	0.295	F(1,20) = 26.86	**0.0002**
5b - CA1	F(1,20) = 27.22	**<0.0001**	F(1,20) = 12.79	0.0019	F(1,20) = 18.35	**<0.0001**
5b - CA2	F(1,20) = 172.9	**<0.0001**	F(1,20) = 0.25	0.620	F(1,20) = 57.73	**<0.0001**
5b - CA3	F(1,20) = 1.15	0.297	F(1,20) = 4.53	**0.046**	F(1,20) = 1.96	0.153
5b - BLA	F(1,20) = 27.04	**<0.0001**	F(1,20) = 17.26	**0.0005**	F(1,20) = 23.85	**<0.0001**
5c - CA1	F(1,20) = 32.41	**<0.0001**	F(1,20) = 14.67	**0.0010**	F(1,20) = 16.13	**<0.0001**
5c - CA2	F(1,20) = 37.28	**<0.0001**	F(1,20) = 0.13	0.723	F(1,20) = 12.51	**<0.0001**
5c - CA3	F(1,20) = 11.34	**0.0031**	F(1,20) = 9.10	**0.0068**	F(1,20) = 6.82	**0.0024**
5c - BLA	F(1,20) = 66.44	**<0.0001**	F(1,20) = 44.77	**<0.0001**	F(1,20) = 16.82	**0.0006**
5d - CA1	F(1,20) = 60.64	**<0.0001**	F(1,20) = 52.70	**<0.0001**	F(1,20) = 19.60	**0.0005**
5d - CA2	F(1,20) = 7.80	**0.0112**	F(1,20) = 2.51	0.1288	F(1,20) = 2.60	0.1247
5d - CA3	F(1,20) = 6.01	**0.0236**	F(1,20) = 0.45	0.5093	F(1,20) = 7.62	**0.0099**
5d - BLA	F(1,20) = 0.47	0.5006	F(1,20) = 3.51	0.0756	F(1,20) = 0.70	0.5761
5e - CA1	F(1,20) = 67.38	**<0.0001**	F(1,20) = 69.18	**<0.0001**	F(1,20) = 45.93	**<0.0001**
5e - CA2	F(1,20) = 7.36	**0.0134**	F(1,20) = 4.53	**0.0459**	F(1,20) = 4.41	**0.0155**
5e - CA3	F(1,20) = 7.64	**0.0120**	F(1,20) = 1.83	0.1914	F(1,20) = 15.21	**<0.0001**
5e - BLA	F(1,20) = 0.25	0.6259	F(1,20) = 6.58	**0.0185**	F(1,20) = 2.41	0.0971
5f - CA1	F(1,20) = 72.85	**<0.0001**	F(1,20) = 62.04	**<0.0001**	F(1,20) = 45.81	**<0.0001**
5f - CA2	F(1,20) = 8.30	0.0092	F(1,20) = 3.87	0.0632	F(1,20) = 6.48	**0.0030**
5f - CA3	F(1,20) = 5.64	**0.0277**	F(1,20) = 8.93	**0.0073**	F(1,20) = 6.52	**0.0030**
5f - BLA	F(1,20) = 23.57	**<0.0001**	F(1,20) = 4.57	**0.0451**	F(1,20) = 10.36	**0.0003**
6a - CM	F(1,16) = 16.34	**0.0009**	F(1,16) = 20.75	**0.0003**	F(1,16) = 20.75	**0.0003**
6a - FP	F(1,16) = 11.97	**0.0032**	F(1,16) = 5.70	**0.0297**	F(1,16) = 8.87	**0.0089**
6b - CM	F(1,16) = 10.70	**0.0048**	F(1,16) = 10.59	**0.0050**	F(1,16) = 10.59	**0.0050**
6b - FP	F(1,16) = 7.75	**0.0133**	F(1,16) = 0.51	0.4872	F(1,16) = 2.35	0.1446
6c - CM	F(1,16) = 24.38	**0.0001**	F(1,16) = 35.45	**<0.0001**	F(1,16) = 25.70	**0.0001**
6c - FP	F(1,16) = 5.45	**0.0330**	F(1,16) = 28.49	**<0.0001**	F(1,16) = 36.52	**<0.0001**
6d - CM	F(1,16) = 156.1	**<0.0001**	F(1,16) = 0.01	0.9190	F(1,16) = 6.99	**0.0177**
6d - FP	F(1,16) = 74.61	**<0.0001**	F(1,16) = 0.01	0.9141	F(1,16) = 1.08	0.3153

## 3 Results

### 3.1 Maternal social stress increases circulating corticosterone in the mother but not the fetuses

**Fig. 1:**
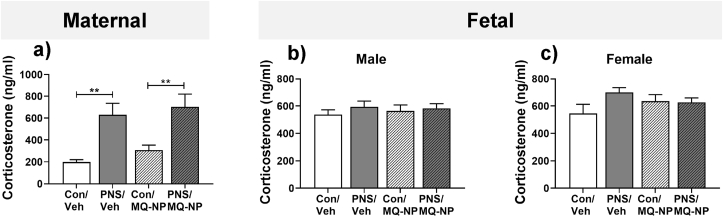
**Maternal social stress increases plasma corticosterone concentrations in the mother but not the fetuses.** Concentrations of corticosterone were measured in maternal plasma (a), male fetal plasma (b) and female fetal plasma (c) collected on GD 20, from unstressed dams (Con) or from dams exposed to 10 min social stress for 5 consecutive days during the last week of pregnancy (PNS). Pregnant rats received an intravenous injection of either vehicle (veh) or MitoQ-NP (MQ-NP) prior to the exposure. **p < 0.01 vs respective control group (two-way ANOVA followed by Student-Newman-Keuls post-hoc test). n = 7/group.

There was a significant main effect of stress exposure, but not drug treatment on plasma corticosterone concentrations in the dams (Fig. 1a, Table 1). Circulating corticosterone concentrations were significantly greater in mothers exposed to repeated social stress during pregnancy, compared with non-stressed mothers, irrespective of drug treatment (Fig. 1a, Table 1). In contrast, neither maternal stress nor drug treatment had any significant effect on plasma corticosterone concentrations in the male (Fig. 1b, Table 1) or female fetuses (Fig. 1c, Table 1).

### 3.2 Maternal social stress increases oxidative stress in the placenta but not the fetal brain

**Fig. 2:**
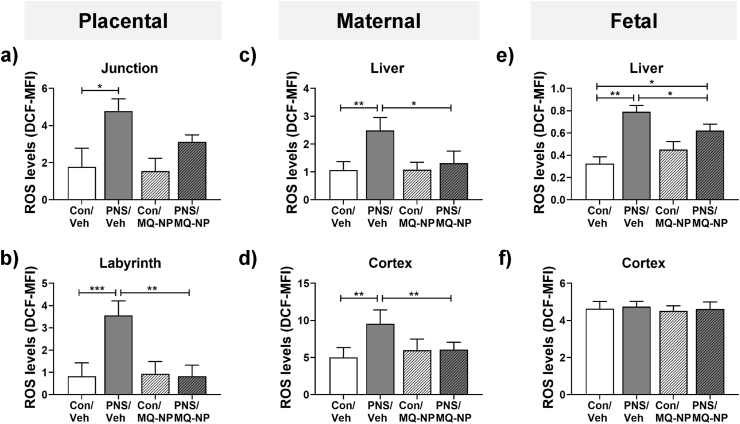
**Oxidative stress levels in the mother and fetuses following maternal social stress with and without maternal antioxidant treatment.** Mean fluorescence intensity (MFI) of dihydrodichlorofluorescein (DCF), a measure of reactive oxygen species (ROS) levels and a marker of oxidative stress, were quantified in the junctional (a) and labyrinth (b) zones of the placenta, maternal liver (c) and cerebral cortex (d), as well as in the fetal liver (e) and cerebral cortex (f). Tissues were collected on gestational day (GD) 20 from unstressed dams (Con) or from dams exposed to 10 min of social stress for 5 consecutive days during the last week of pregnancy (PNS). Pregnant rats received an intravenous injection of either vehicle (veh) or MitoQ-NP (MQ-NP) prior to the exposure. MFI, mean fluorescence intensity. *p < 0.05, **p < 0.01, ***p < 0.001 between groups indicated (two-way ANOVA followed by Tukey's post-hoc test). n = 3/group for maternal, fetal and placental samples.

Next, we examined whether exposure to psychosocial stress in pregnancy induces oxidative stress. Following maternal social stress, ROS levels were significantly greater in the junctional and labyrinth zones of the placenta (Fig. 2a and b, Table 1), maternal liver and cerebral cortex (Fig. 2c and d, Table 1), and fetal liver (Fig. 2e, Table 1). In contrast, ROS levels in the fetal cerebral cortex were not significantly altered by maternal social stress (Fig. 2f, Table 1).

Maternal antioxidant treatment (MitoQ-NP) prevented the increase in ROS in the placenta (Fig. 2a and b, Table 1), maternal liver (Fig. 2c, Table 1), maternal cerebral cortex (Fig. 2d, Table 1) and fetal liver (Fig. 2e, Table 1), but had no effect on ROS levels in the fetal cerebral cortex (Fig. 2f, Table 1).

### 3.3 Maternal antioxidant prevents anxiety-like behavior in male prenatally stressed offspring

**Fig. 3:**
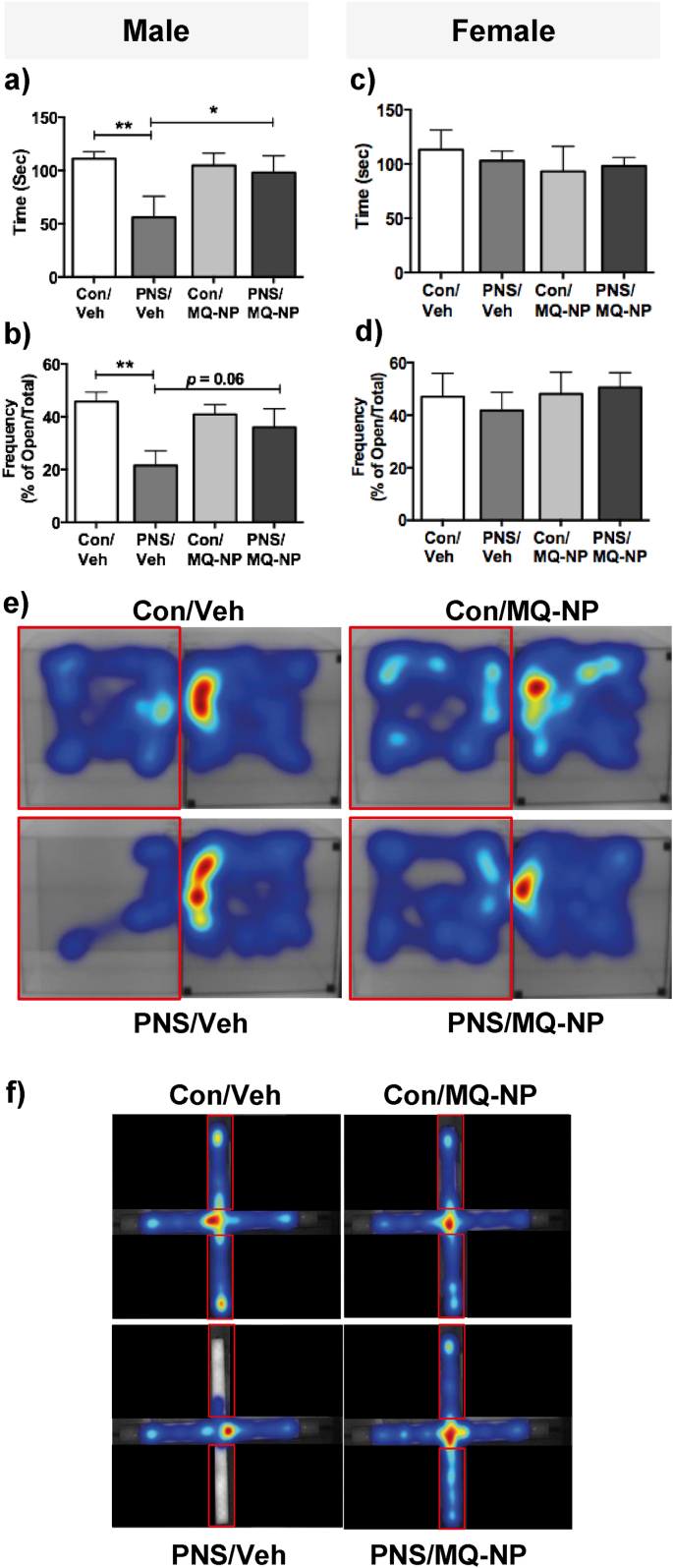
**Effects of prenatal stress and maternal antioxidant treatment on anxiety-like behavior in the offspring.** The male and female offspring of stressed and non-stressed dams, treated with vehicle or MitoQ-NP, were tested for anxiety-like behavior using the light-dark box (LDB) and the elevated plus maze (EPM). Time spent in the light compartment of the LDB (a, c) and the percentage of open arm entries on the EPM (b, d) was recorded. *p < 0.05, **p < 0.01 between groups indicated (two-way ANOVA followed by Student-Newman Keuls post-hoc test). n = 8/group. Heat maps illustrating movement of male rats in the LDB (e) and the EPM (f). Red boxes represent the light compartment and open arms of the LDB and EPM, respectively. (For interpretation of the references to colour in this figure legend, the reader is referred to the Web version of this article.)

We next hypothesized that by preventing the maternal psychosocial stress-induced increase in ROS in the placenta using an antioxidant, we could prevent the development of the anxious phenotype we previously reported in the male offspring (Brunton and Russell, 2010). There was a significant main effect of prenatal stress on behavior in the light-dark box (Table 1) and on the elevated plus maze (Table 1) in the male rats. Male prenatally stressed offspring displayed increased anxiety-like behavior compared with control males, reflected by spending significantly less time in the light compartment of the light-dark box (Fig. 3a, e; Suppl. Fig. S1; Table 1) and making fewer open arm entries on the elevated plus maze (Fig. 3b, f; Suppl. Fig. S1; Table 1). This was not a result of altered locomotion in the prenatally stressed males (Suppl. Fig. S1). In contrast, maternal social stress had no significant effect on anxiety-like behavior in either test in the female offspring (Fig. 3c and d; Table 1), consistent with our previous findings (Brunton and Russell, 2010).

Maternal administration of MitoQ-NP prior to social stress exposure prevented the development of anxiety-like behavior in the male prenatally stressed offspring (Fig. 3a, b, e, f) and had no effect in the female offspring (Fig. 3c and d).

### 3.4 Maternal antioxidant treatment prevents sex-specific neurobiological changes in male prenatally stressed offspring

Given dendrite pathology is reported in psychiatric disorders (Martinez-Tellez et al., 2009; Mychasiuk et al., 2012; Glausier and Lewis, 2013; Curran et al., 2017) and disrupted inhibitory signalling in limbic brain structures is associated with anxiety behavior (Laloux et al., 2012; Liang et al., 2016; Zou et al., 2016), we investigated the impact of maternal stress on dendrite length and the number of parvalbumin-positive (PV+) neurons in the basolateral amygdala and hippocampus of the offspring.

**Fig. 4:**
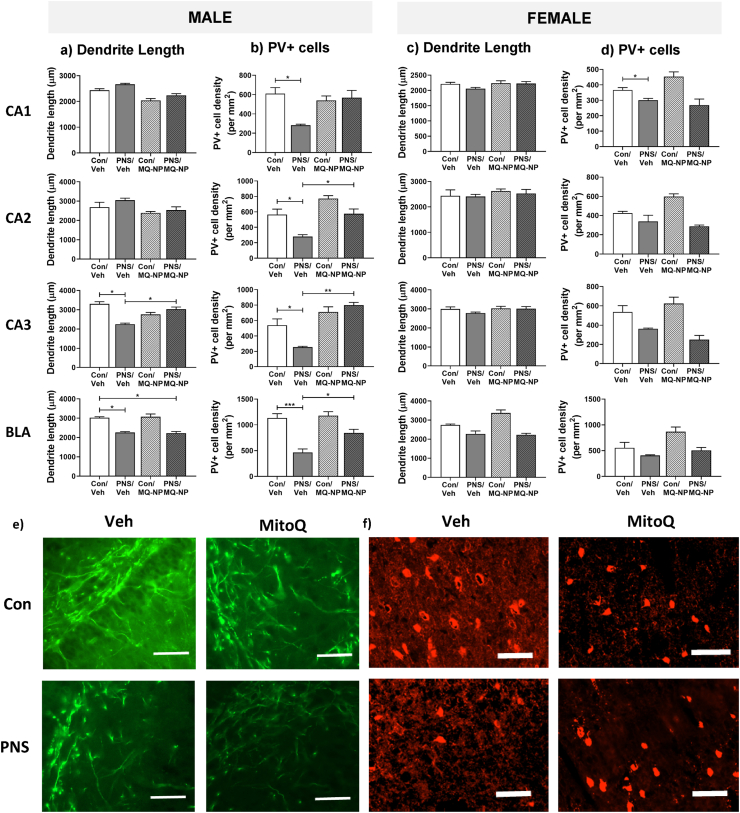
**Neuroanatomical effects of maternal social stress and antioxidant treatment in the offspring.** Brain tissue from rats (n = 6/group), born to stressed or non-stressed mothers administered either vehicle or MitoQ-NP during pregnancy, were processed using immunohistochemistry. Dendrite lengths (a, c) and parvalbumin-positive (PV+) cell numbers (b, d) were quantified in the CA1, CA2, CA3 hippocampal subfields and in the basolateral amygdala (BLA) of male (a, b) and female (c, d) offspring. *p < 0.05, **p < 0.01, ***p < 0.001 (two-way ANOVA followed by Tukey's post-hoc test). Representative images of MAP2 immunostaining in the CA3 hippocampal subfield of male rats (e; scale bar = 200 μm). Representative images of parvalbumin immunostaining in the BLA of male rats (f; scale bar = 100 μm).

A significant reduction in dendrite length in the CA3 region of the hippocampus and in the basolateral amygdala was observed in male offspring born to mothers exposed to social stress (Fig. 4a and e; Table 1). Moreover, there was a significant loss of immunoreactive PV+ neurons across all the hippocampal subfields and in the basolateral amygdala of male prenatally stressed offspring, compared with controls (Fig. 4b and f; Table 1).

In contrast, prenatal stress did not affect dendrite length in any of the examined brain regions (Fig. 4c; Table 1) in the female offspring. A significant reduction in the density of PV+ neurons, compared to female controls, was observed only in the hippocampal CA1 region (Fig. 4d; Table 1).

Maternal administration of MitoQ-NP significantly prevented the prenatal stress-induced reduction in dendritic length in the hippocampal CA3 region (Fig. 4a; Table 1), and the loss of PV+ neurons in the hippocampus and basolateral amygdala (Fig. 4b; Table 1) of the male offspring. The same treatment had no effect on dendrite length or on the number of PV+ neurons in female offspring (Fig. 4c and d; Table 1). Neither prenatal stress nor MitoQ-NP treatment altered the overall density of neurons in male or female offspring in any of the regions examined (Suppl. Fig. S2).

**Fig. 5:**
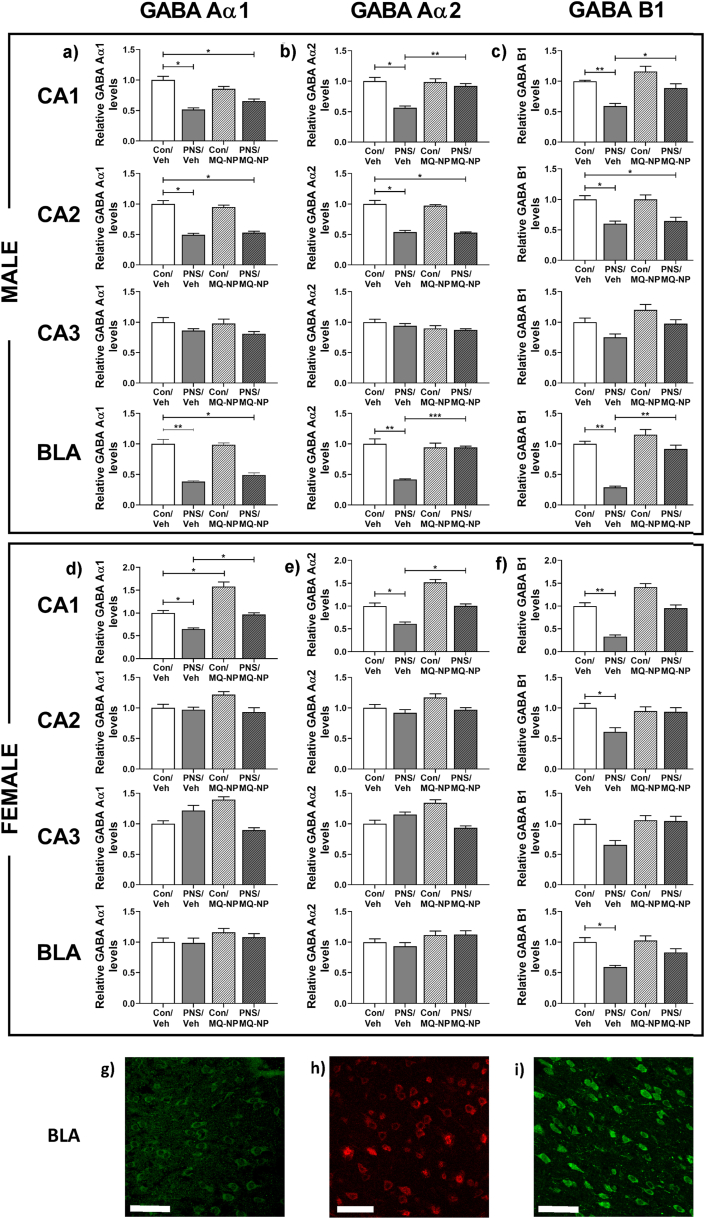
**Effects of maternal social stress and antioxidant treatment on GABA receptor subunit expression in the offspring hippocampus and amygdala.** Immunoreactivity for GABA receptor subunits was assessed in the brains of offspring (n = 6/group) born to mothers exposed to social stress or undisturbed during pregnancy, and pre-treated with either vehicle or MitoQ-NP. GABA Aα1 (a, d), GABA Aα2 (b, e) and GABA B1 (c, f) subunit expression was quantified in the CA1, CA2, CA3 hippocampal subfields and in the basolateral amygdala (BLA) of both male and female offspring. *p < 0.05, **p < 0.01, ***p < 0.001 (two-way ANOVA followed by Tukey's post-hoc test). Representative images of immunostaining for GABA Aα1 (g), GABA Aα2 (h) and GABA B1 (i) in the BLA of a control/veh male rat. Scale bars = 100 μm in each case.

Immunoreactivity for the GABA receptor subunits Aα1, Aα2 and B1 was significantly lower in the CA1 and CA2 hippocampal subfields and in the basolateral amygdala of prenatally stressed male offspring, compared to control males (Fig. 5a–c, g-i; Table 1). In female prenatally stressed offspring, GABA Aα1 and Aα2 levels were reduced in the CA1 region (Fig. 5d and e; Table 1); while GABA B1 immunoreactivity was significantly lower in the CA1 and CA2 hippocampal subfields and in the basolateral amygdala (Fig. 5f; Table 1).

Maternal administration of MitoQ-NP prevented the prenatal stress-induced reduction in GABA Aα2 and B1 in both the CA1 and basolateral amygdala in the male offspring (Fig. 5b and c; Table 1) and the reduction of GABA Aα1 and Aα2 in the CA1 region of the female offspring (Fig. 5d and e; Table 1).

### 3.5 Exposing neuronal cultures to fetal plasma or placenta-conditioned medium from stressed pregnancies mimics the neurobiological effects of prenatal stress

**Fig. 6:**
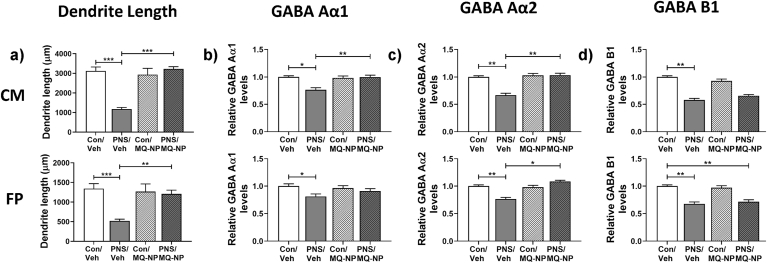
**Effects of treating cortical cultures with fetal plasma or placental-conditioned medium from stressed and non-stressed pregnancies.** Fetal plasma (FP) and culture medium conditioned by placentae (CM) collected on gestational day 20 from stressed or non-stressed mothers administered either vehicle or MitoQ-NP during pregnancy were applied to cortical cultures. Dendrite lengths (a) and expression of GABA Aα1 (b), GABA Aα2 (c) and GABA B1 (d) subunits were quantified. *p < 0.05, **p < 0.01, ***p < 0.001 (two-way ANOVA followed by Tukey's post-hoc test). Group numbers: n = 5/group.

We next tested whether the placenta secretes ‘factors’ into the fetal circulation that alter fetal brain development. Application of placenta-conditioned medium or fetal plasma from prenatally stressed rats to neuronal cultures resulted in a significant reduction in dendritic length (Fig. 6a; Table 1). Maternal MitoQ-NP treatment prevented these stress-induced effects on dendrite length (Fig. 6a; Table 1). The total density of neurons was not affected by any of the treatments (Suppl. Fig. S3). The paucity of PV+ cells in cortical cultures meant no meaningful counts could be made.

Immunoreactivity for GABA receptor subunits Aα1, Aα2 and B1 was significantly lower in neuronal cultures exposed to either placental-conditioned medium or fetal plasma from stressed rats (Fig. 6b–d; Table 1). Maternal MitoQ-NP treatment was successful in preventing the reduced levels of GABA Aα1 and Aα2 in neuronal cultures exposed to conditioned medium (Fig. 6b and c; Table 1) and levels of Aα2 in cultures exposed to fetal plasma (Fig. 6c; Table 1).

### 3.6 microRNAs are differentially secreted from the placenta in response to maternal social stress

**Fig. 7:**
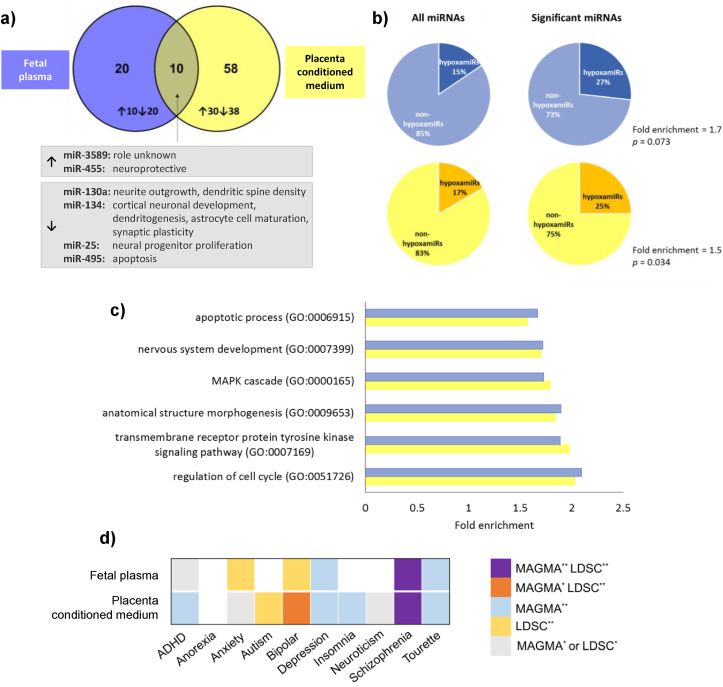
**Circulating microRNAs in fetal plasma and placenta-conditioned medium following maternal social stress.** Pooled fetal plasma (blue) and placenta-conditioned culture medium (yellow), collected from pregnant rats exposed to social stress and treated with vehicle, was assessed for levels of microRNAs. (a) Differentially abundant microRNAs following prenatal stress were compared between fetal plasma and placental conditioned medium. Ten microRNAs showed significant differential abundance both in fetal plasma and placenta-conditioned medium from stressed rats; two of these microRNAs were significantly upregulated and four significantly downregulated in both plasma and conditioned medium. The six microRNAs that were similarly up-or down-regulated in both fetal plasma and conditioned medium are listed with their known functions. (b) Significant differentially abundant microRNAs were enriched for hypoxamiRs, both in fetal plasma (top) and placenta conditioned medium (bottom). (c) Gene ontology analysis was performed on the predicted targets of differentially abundant microRNAs. The top 6 significant gene ontology terms are listed, along with fold enrichment of the term among the predicted targets. (d) Gene set association analysis across neuropsychiatric traits. Two classical approaches, MAGMA and LDSC, have been used to test enrichment in GWAS signals of the predicted targets of the differentially abundant microRNAs in fetal plasma or placenta-conditioned medium following prenatal stress. The heatmap colours indicate if a significant association is obtained by one or both methods at multiple test correction significance threshold (**) or nominally significant threshold (p ≤ 0.05) (*). Of particular interest are those associations that were significant following correction for multiple comparisons using either method (orange, blue and yellow) or both methods (purple). Group numbers: n = 3/group for plasma and conditioned medium (each from a different litter). (For interpretation of the references to colour in this figure legend, the reader is referred to the Web version of this article.)

Finally, we sought potential factors released by the placenta that may mediate the effects of maternal social stress on the fetal brain. microRNAs are small post-transcriptional regulators that play a role in neurodevelopment and in mood disorders, including anxiety (O'Connor et al., 2012; Sun and Shi, 2015). We detected differentially abundant microRNAs in placenta-conditioned medium and in fetal plasma, collected from stressed pregnancies compared to unstressed pregnancies (Fig. 7a; Suppl. Dataset 1); with 10 microRNAs showing significant differential abundance in both fetal plasma and placenta-conditioned medium from stressed rats (Fig. 7a). Maternal MitoQ-NP administration prevented stress-induced changes in 12/68 microRNAs in conditioned medium and in 4/30 microRNAs in fetal plasma (Table 2; Suppl. Dataset 1).

**Table 2:** miRNAs that were both significantly upregulated by prenatal stress exposure and significantly downregulated by maternal MitoQ-NP treatment, or vice versa, in fetal plasma (FP) or placenta-conditioned medium (CM). To assess the effect of prenatal stress on miRNA abundance, log2-fold change (log2FC) and *p* values are presented for samples collected from vehicle-treated rats exposed to prenatal stress, relative to vehicle-treated controls. To quantify the effect of MitoQ-NP treatment, log2FC and *p* values were calculated for samples collected from MitoQ-NP-treated prenatally stressed rats, compared to vehicle-treated rats exposed to prenatal stress.

		Effect of prenatal stress		Effect of MitoQ-NP	
Sample	miRNA	log2FC	*p* value	log2FC	*p* value
**FP**	miR-376a	1.09	0.031	−0.43	0.038
	miR-455	0.50	0.048	−0.43	0.018
	miR-144	0.45	0.031	−1.11	<0.001
	miR-206	−1.06	0.007	0.63	0.001
**CM**	miR-196b	1.08	<0.001	−0.52	0.002
	let-7c	0.76	<0.001	−0.50	0.011
	miR-101a	0.67	0.009	−0.64	0.003
	miR-29b	0.65	<0.001	−0.61	<0.001
	miR-33	0.58	0.008	−0.40	0.046
	let-7f	0.57	<0.001	−0.52	0.005
	miR-338	0.55	0.024	−0.80	<0.001
	let-7e	0.54	<0.001	−0.33	0.034
	let-7a	0.48	<0.001	−0.44	0.010
	miR-466c	0.42	0.001	−0.39	0.023
	miR-434	−0.29	0.013	0.37	0.030
	miR-423	−0.32	0.017	0.60	<0.001

microRNAs significantly altered by prenatal stress included a higher proportion of hypoxamiRs, hypoxia-regulated microRNAs, and this enrichment was significant for microRNAs in conditioned medium (Fig. 7b). Gene ontology analysis revealed that the predicted targets of the microRNAs significantly altered by maternal stress were enriched for processes relating to nervous system development and signalling pathways, both for conditioned medium and fetal plasma (Fig. 7c). Furthermore, risk genes for several psychiatric disorders, including anxiety, depression, bipolar disorder and schizophrenia were enriched among these predicted microRNA targets (Fig. 7d).

## 4 Discussion

Here we investigated a role for oxidative stress in mediating the adverse impact of maternal social stress on the offspring's brain and behavior. We demonstrate for the first time that maternal social stress during pregnancy induces oxidative stress in the placenta. Moreover, prenatal stress-induced anxiety-like behavior in the male, but not female offspring, and this was associated with a sex-specific reduction in dendrite length and markers of GABAergic signalling in limbic brain regions. Critically these behavioral and neurobiological phenotypes were prevented by maternal administration of an antioxidant, prior to stress exposure. Furthermore, many of the neural effects were mimicked in neuronal cultures by application of placenta-conditioned medium or fetal plasma from stressed pregnancies, but not if the dams were treated with the antioxidant. Finally, we demonstrated that the pattern of microRNAs secreted by the placenta is altered following maternal social stress, highlighting a potential role for microRNAs in mediating the effects of maternal social stress on the fetal brain.

Stress-induced increases in maternal glucocorticoids have been proposed to mediate the programming effects of prenatal stress on the fetal brain; however, limited evidence supports this assertion (Barbazanges et al., 1996; Rakers et al., 2017). Here, pregnant rats exposed to social stress during late pregnancy showed a marked increase in corticosterone secretion; however corticosterone concentrations in the fetal circulation were unaffected, indicating that maternal corticosterone is unlikely to directly program the fetal brain. Rather our data suggest that oxidative stress may play a role. Whether maternal glucocorticoids contribute to fetal programming indirectly by initiating a cascade of downstream events, for example via inducing oxidative stress in the placenta, remains to be elucidated.

Previous research indicates the placenta plays a key role in fetal programming of psychiatric disorders that result from adverse events during pregnancy (O'Donnell et al., 2009; Brunton and Russell, 2011; Bronson and Bale, 2016). In accordance, we demonstrate that maternal social stress induces oxidative stress in the placenta, highlighting the potential significance of the placenta in mediating the effects of stress on the offspring. Maternal administration of a nanoparticle-bound antioxidant MitoQ-NP [that enters the placenta but does not cross it (Phillips et al., 2017)] reduced placental and maternal oxidative stress and critically prevented sex-specific behavioral and neurobiological changes in the offspring of mothers exposed to social stress. Specifically, male, but not female rats, showed increased anxiety-like behavior following exposure to prenatal stress, while dendrite lengths and the number of PV+ neurons were reduced, almost selectively, in the hippocampus and basolateral amygdala of the male offspring. The hippocampus and amygdala are well established to be involved in regulating anxiety behavior and stress responses (McEwen, 2016; Sharp, 2017; Zhang et al., 2018) and structural and neurochemical changes within these structures have been reported following prenatal stress (Hayashi et al., 1998; Kraszpulski et al., 2006; Laloux et al., 2012; Mychasiuk et al., 2012; Bock et al., 2014; Soares-Cunha et al., 2018).

Furthermore, male prenatally stressed rats showed reductions in GABA receptors in the CA1 and CA2 hippocampal subfields, and in the BLA, while changes in female offspring were less pronounced or completely absent. These results support a role for a dysfunction in the GABAergic system in the offspring as a result of maternal social stress, which is noteworthy as impaired GABAergic signalling is implicated in the pathophysiology of psychiatric disorders, including anxiety (Nikolaus et al., 2010; Fine et al., 2014; Tovote et al., 2015; Selten et al., 2018).

The sex-specific changes in the offspring hippocampus and basolateral amygdala in response to maternal social stress may underlie the sex-dependent behavioral phenotypes in the offspring. Why these brain regions are particularly susceptible to stress-induced changes in male, but not female rats is unclear. Differences in sex steroids may render certain brain regions more vulnerable to prenatal stress in males versus females (Sze and Brunton, 2019), however, differences in gene expression or epigenetic profiles are also likely to play a role (McCarthy et al., 2009; Brunton et al., 2011; Ratnu et al., 2017). Exposure to repeated restraint stress during the last week of gestation blocks the prenatal testosterone surge in male fetuses, and is associated with abnormal sexual behaviour in adulthood (Ward and Weisz, 1980, 1984); hence, maternal social stress could potentially disrupt the organisational effects of steroids on the brain during prenatal development, leading to altered activational effects of steroids and behavior in later life. Furthermore, sex-specific differences in the placenta, particularly differences in gene expression (Mueller and Bale, 2008; Osei-Kumah et al., 2011; Gabory et al., 2013; Howerton et al., 2013; Howerton and Bale, 2014), epigenetic profiles (Hamdan et al., 2009; Nugent et al., 2018), and metabolism (O'Connell et al., 2013), could render male and female placentae differentially responsive to maternal insults (Clifton, 2010; Hsiao and Patterson, 2012). Based on our neurological and behavioral findings, it could be predicted that sex differences exist in the levels of placental oxidative stress induced by maternal social stress, with male fetuses being more vulnerable than females; however this may not be the case. Indeed, we have recently shown that the ROS, superoxide, is increased to a similar extent in the placenta of male and female foetuses whose mothers were exposed to hypoxia in late pregnancy, despite the male fetuses seemingly being more susceptible to the adverse effects of maternal hypoxia (Ganguly et al., 2019). Moreover, we did not detect any change in ROS in the fetal brain in response to maternal social stress. Together these data suggest that oxidative stress does not act directly on the offspring's brain to induce the changes observed, but instead it may act as a mediator that triggers sex-dependent downstream effects.

Remarkably, MitoQ-NP treatment also prevented prenatal stress-induced reductions in dendrite lengths, the number of PV+ neurons and GABA receptor subunit expression in most of the hippocampal subfields and in the basolateral amygdala of male rats. These findings are consistent with our previous studies where antioxidant treatment prevented a reduction in dendrite lengths induced by gestational hypoxia or preeclampsia (Phillips et al., 2017; Scott et al., 2018). The present data support a role for oxidative stress in mediating the effects of maternal psychosocial stress on the offspring's brain and raise the possibility of treating placental oxidative stress (without affecting the fetus directly) to prevent neurodevelopmental programming by maternal psychological stress. Nevertheless, any preventative treatment needs to be cognisant of the sex differences in prenatal stress effects.

Application of fetal plasma or placenta-conditioned culture medium from stressed pregnancies to cultured neurons mimicked many of the neurobiological effects observed in the prenatally stressed offspring *in vivo*. Due to the low volume of fetal plasma collected, samples were pooled across litters, meaning comparisons could not be made between sexes. Despite this limitation, the data suggest that molecules secreted from the placenta into the fetal circulation can affect fetal neurons and is consistent with our hypothesis that factors released from the placenta may mediate the effects of maternal insults during pregnancy on the developing fetal brain (Bhabra et al., 2009; Sood et al., 2011; Curtis et al., 2014; Phillips et al., 2017; Hawkins et al., 2018; Scott et al., 2018). Given the effects observed in the neuronal cultures largely mirrored the neurobiological effects observed in the male offspring, it may be the case that there is a dominance of factors released by male placentae into the male fetal blood. Alternatively, there may be a sex difference in the way in which these placental factors impact the developing brain, with males potentially being more vulnerable. These issues and the identity of the placental factors requires further study, however it may involve differential release of microRNAs from the placenta.

We identified several microRNAs secreted by the placenta in response to maternal stress. Predicted targets of the differentially abundant microRNAs were enriched for genes involved in neurodevelopment. Genome-wide association studies of psychiatric disorders have identified genes that are associated with increased risk and therefore likely to contribute to disease aetiology. Predicted targets of the microRNAs identified in this study were enriched for risk genes for several psychiatric disorders, including anxiety and depression. These analyses suggest that the microRNAs that are differentially released from the placenta following maternal stress could target genes important for neurodevelopment and psychiatric disease.

Extracellular microRNAs can act as long-distance messengers. They can be taken up by cells, including neurons, where they fulfil their regulatory function (Scott, 2017). Furthermore, microRNAs have the potential to cross the blood-brain barrier and enter the brain, when encapsulated within extracellular vesicles (Alvarez-Erviti et al., 2011). While the potential of microRNAs as mediators of maternal stress effects on the fetal brain needs further investigation; we hypothesize that following chronic maternal social stress, extracellular microRNAs released into the fetal circulation from the oxidatively stressed placenta may cross the fetal blood-brain barrier, where they can be taken up by neurons and transiently affect the expression of key developmental genes. Given the distinct sex differences in the neurobiological and behavioral outcomes observed in the offspring following maternal stress, we further predict that sex differences likely exist in the abundance and/or impact of these extracellular microRNAs on the developing brain.

The prenatal period is critical for healthy development; however, very little in the way of preventative treatment exists for cases when the prenatal environment is suboptimal, e.g. during periods of chronic maternal stress. The present study highlights for the first time the importance of oxidative stress in the placenta – and factors released from the placenta into the fetal circulation – in mediating the sex-specific behavioral and neurobiological changes in the offspring following maternal social stress during pregnancy. While further assessment of maternal antioxidant treatment in pregnancy will be required, especially in light of the observed offspring sex differences, the present study highlights the potential for drugs such as MitoQ-NP to be used as preventative treatment with long-term implications for offspring mental health.

## CRediT authorship contribution statement

**H. Scott:** Validation, Formal analysis, Investigation, Writing - original draft, Writing - review & editing, Visualization. **T.J. Phillips:** Validation, Formal analysis, Investigation, Writing - original draft, Visualization. **Y. Sze:** Validation, Formal analysis, Investigation, Writing - original draft, Visualization. **A. Alfieri:** Investigation. **M.F. Rogers:** Formal analysis. **V. Volpato:** Formal analysis, Visualization. **C.P. Case:** Conceptualization, Methodology, Supervision, Funding acquisition. **P.J. Brunton:** Conceptualization, Methodology, Investigation, Writing - original draft, Writing - review & editing, Supervision, Funding acquisition.

## Declaration of competing interest

HS and TJP have previously consulted for Placentum Ltd. The University of Bristol has filed a patent application for the nanoparticle formulation used in this study and its application to pregnancy-related diseases. The other authors report no conflicts of interest.
