# Supplementary material for: Maternal antioxidant treatment prevents the adverse effects of prenatal stress on the offspring's brain and behavior
Source: Neurobiol Stress. 2020 Nov 29;13:100281. doi: 10.1016/j.ynstr.2020.100281 (PMC7739187; doi:10.1016/j.ynstr.2020.100281)
Supplement: Multimedia component 2 [file mmc2.pdf]

SUPPLEMENTARY MATERIAL

Maternal antioxidant treatment prevents the adverse effects of prenatal stress on the offspring's brain and behavior

Scott H., Phillips T.J., Sze Y., Alfieri A., Rogers M.F., Volpato V., Case C.P. & Brunton P.J.

Figure S1

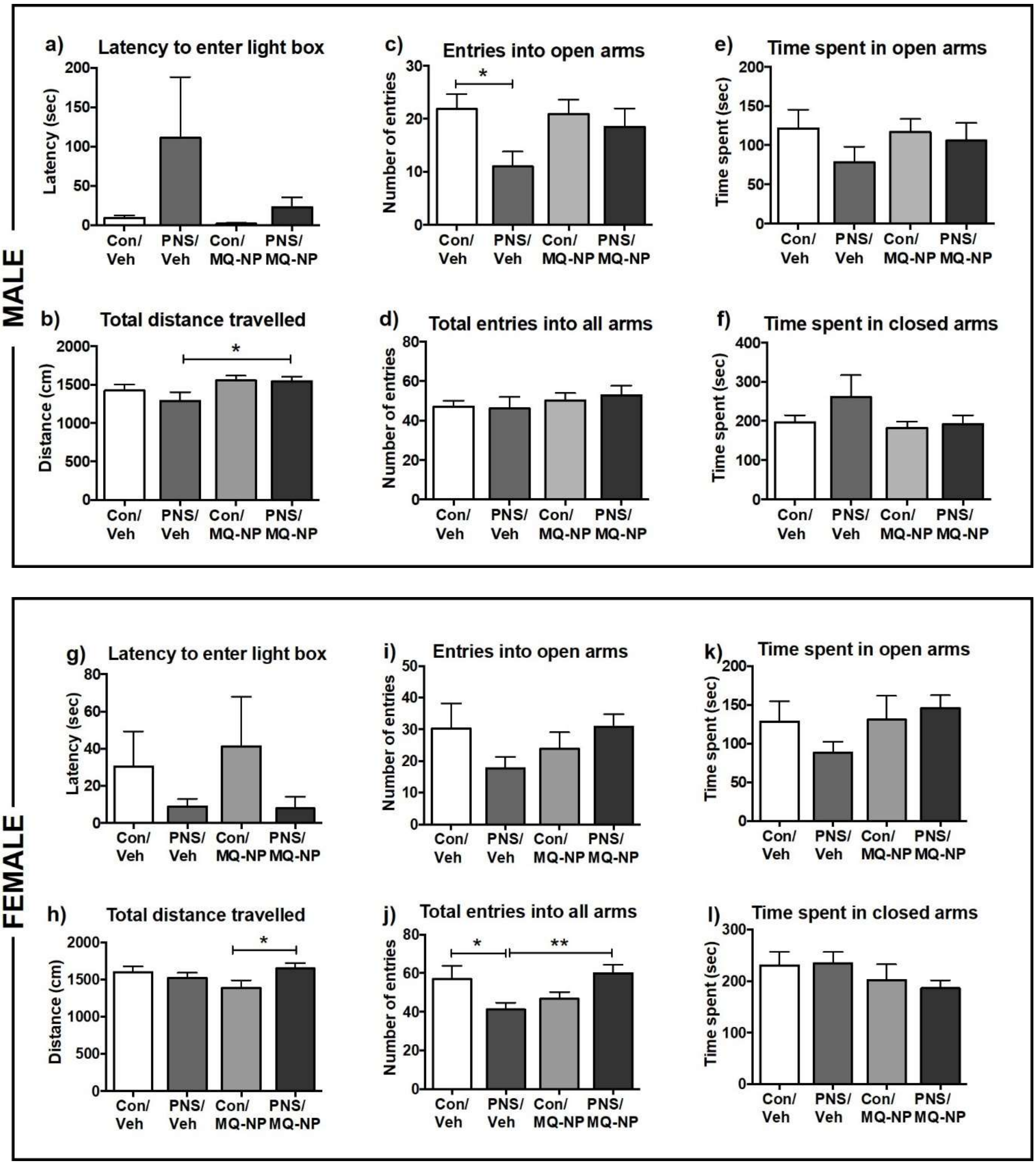

Supplementary Figure S1 | Effects of prenatal social stress and maternal antioxidant treatment on anxiety-like behavior in the offspring. Male (top panel) and female (bottom panel) offspring of mothers (n = 8/group) from non-stressed pregnancies (Con) or from those exposed to social stress during pregnancy (PNS) and administered either vehicle (Veh) or MitoQ-NP (MQ-NP) were tested in the light-dark box (a, b, g, h) and the elevated plus maze (c-l). Two-way ANOVA revealed no significant effect of prenatal experience on the total distance travelled in the light-dark box in males (indicating locomotion is not affected by PNS); however there was a significant effect of MitoQ-NP on the total distance travelled in the light-dark box in males ($F_{1,27}$ = 5.2, p = 0.031; b) and a significant prenatal stress x MitoQ-NP interaction in females ($F_{1,27}$ = 4.34, p = 0.047; h). On the elevated plus maze, there

was no significant effect of prenatal status or drug treatment on the total number of arm entries (again indicating locomotion is unaffected; d), however there was a significant effect of prenatal stress on the number of entries into the open arms in male rats ($F_{1,27} = 4.82$, $p = 0.037$; c) and a significant stress x drug interaction on total entries into all arms in female rats ($F_{1,27} = 9.24$, $p = 0.005$; j). There was no significant effect of prenatal experience or MItoQ-NP treatment on the total time spent in the open (e, k) or the closed arms (f, l) of the maze in either sex. *p<0.05;**p<0.01.

Figure S2

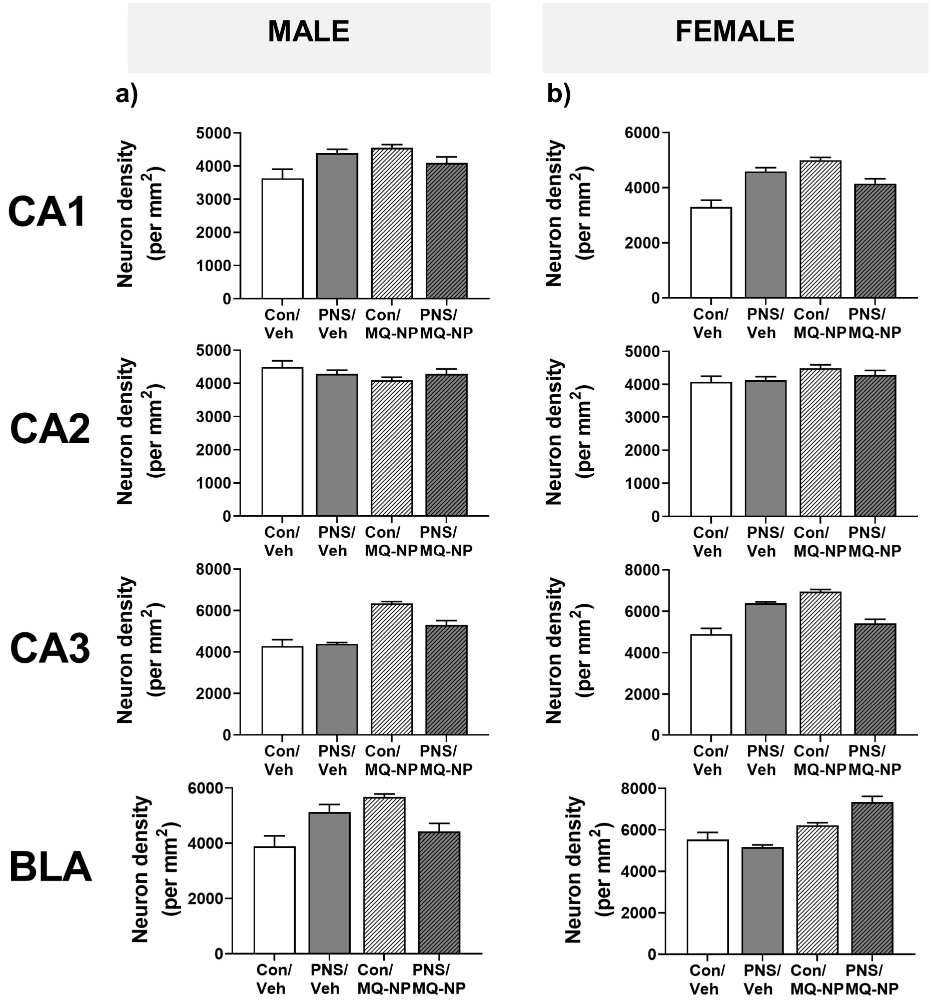

Supplementary Figure S2 | Neuronal densities in the brains of offspring exposed to prenatal social stress and maternal antioxidant treatment. Neuronal counts were assessed in male and female offspring (n = 6/group) exposed to prenatal social stress (PNS) and maternal intravenous injection of vehicle (Veh) or MitoQ-NP (MQ-NP). CA1, CA2 and CA3 regions of the hippocampus and basolateral amygdala (BLA) were analysed. Two-way ANOVA revealed no significant differences between any of the groups. Neither prenatal stress nor maternal MitoQ-NP treatment altered the overall density of neurons in any of the regions examined in either males (a) or females (b).

Figure S3

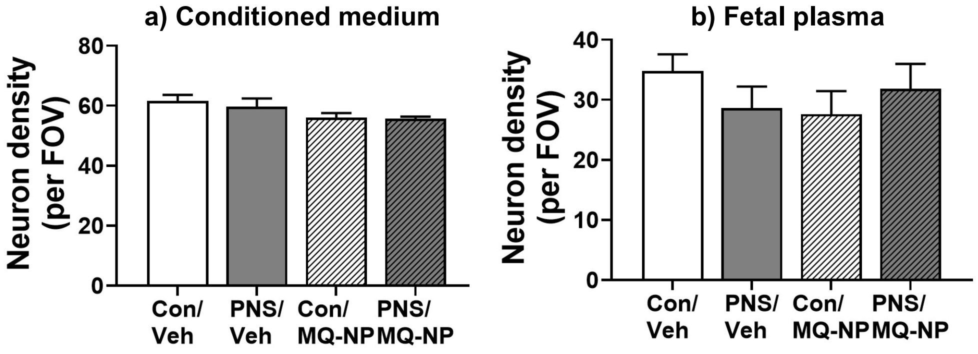

Supplementary Figure S3 | Neuronal densities in cortical cultures exposed to placental conditioned medium and fetal plasma from stressed pregnancies. Culture medium conditioned by placentae (a) and fetal plasma (b) collected on gestational day 20 from dams of the four different treatment groups were applied to cortical cultures and neuronal densities were measured (n = 5/group each from different litters). Two-way ANOVA revealed no significant differences between any of the groups. Neither prenatal stress nor maternal MitoQ-NP treatment altered the overall density of neurons.

Supplementary Dataset 1 | Differentially abundant microRNAs in the fetal plasma and placenta-conditioned medium following exposure to prenatal social stress and maternal antioxidant treatment. Fetal plasma and culture medium conditioned by placentae collected from pregnant rats that were exposed to social stress or were unstressed, with and without maternal administration of MitoQ-NP was analysed for microRNA expression. The data are presented in an Excel file (Supplementary Dataset 1). Log2-fold change and *p* values are presented for differentially expressed genes ($p < 0.05$) between non-stressed controls administered vehicle (Con-veh) and prenatally stressed rats administered vehicle (PNS-veh) in Sheet 1 for fetal plasma (FP) and in Sheet 2 for placenta-conditioned medium (CM). Sheet 3 and 4 show the differentially expressed genes between prenatally stressed rats with vehicle injection (PNS-veh) and those with MitoQ-NP (PNS-MQ) injection for fetal plasma (FP) and placenta-conditioned medium (CM), respectively.
